# Anti-CASPR2 Antibody-Associated Syndrome Presenting With Episodic Ataxia

**DOI:** 10.7759/cureus.59821

**Published:** 2024-05-07

**Authors:** Sofia Lopes, Leonor Francisco, Stefanie Moreira, Sara Varanda, José Manuel Araújo

**Affiliations:** 1 Neurology Department, Unidade Local de Saúde de Braga, Braga, PRT; 2 Neurology Department, Unidade Local de Saúde Alto Minho, Viana do Castelo, PRT

**Keywords:** episodic ataxia, autoimmune cerebellar ataxia, paroxysmal, caspr2 antibodies, antibody-associated

## Abstract

The anti-CASPR2 antibody-associated syndrome is a rare immune-mediated disorder. Most case reports describe neurologic symptoms that include encephalic signs, peripheral nerve hyperexcitability, dysautonomia, or neuropathic pain. We report the case of a 70-year-old man, admitted to the emergency department with complaints of slurred speech and imbalance. Neurological examination was relevant for dysarthria, hyperreflexia, and pancerebellar syndrome. Cranial CT and basic laboratory tests were normal and he spontaneously recovered after 14 hours. Over the next four months, the patient experienced three similar episodes in relation to stressful events (emotional and organic disturbances like prolonged fasting and vaccination). A contrast-enhanced MRI was performed, along with extensive laboratory testing, analysis of cerebrospinal fluid (CSF), paraneoplastic investigation, and next-generation sequencing panel for episodic ataxias. The results revealed oligoclonal bands in the CSF and positive anti-CASPR2 antibodies both in serum and CSF. Three-day-IV- methylprednisolone pulse followed by plasmapheresis and monthly intravenous immunoglobulins was performed with good response. In conclusion, the neurological manifestations that led to the diagnosis of anti-CASPR2 antibody-associated syndrome were intermittent self-limiting episodes of ataxia, often triggered by concurrent stress-inducing factors. This case supports the aim of other authors to add paroxysmal cerebellar ataxia to the spectrum of the anti-CASPR2 antibody syndrome.

## Introduction

The anti-CASPR2 antibody-associated syndrome is a rare neurological disorder characterized by the presence of autoantibodies targeting contactin-associated protein-like 2 (CASPR2), a cell adhesion molecule primarily found in both the peripheral and central nervous systems [1]. These antibodies are part of the anti-voltage-gated potassium channel antibody complex, a biomarker that has been shown to include autoantibodies targeting CASPR2, leucine-rich glioma 1 protein (LGI1), and other unknown antigens [2].

There is a male predominance, as shown by a systematic review of 667 patients, where sixty-nine percent were male with a median age of 54 years (IQR 39-65.5) [3]. The majority of reported cases do not seem to be related to an underlying neoplasm. In cases where it is, thymoma appears to be the most common neoplasm (21.8%) [3].

The clinical presentation can be diverse, ranging from cerebral symptoms (cognition and epilepsy) to peripheral nerve hyperexcitability and neuropathic pain. Autonomic dysfunction and sleep disturbances have also been reported. Among a cohort of 38 patients with anti-CASPR2 antibodies (where twenty presented with encephalitis and seventeen had neuromyotonia or Morvan syndrome, among other diagnoses), five patients identified retrospectively had transient symptoms suggestive of cerebellar impairment. All these patients had a presentation of autoimmune encephalitis with prominent seizures and amnesia. Three patients also developed mild permanent cerebellar symptoms, either concurrently with or after the onset of paroxysmal ataxia [4]. In larger cohorts, cerebellar symptoms have an estimated prevalence of 14% in patients with anti-CASPR2 antibodies [3].

Early recognition of symptoms is crucial for timely intervention and management. Diagnosis typically involves a combination of clinical evaluation, neuroimaging studies, and antibody testing, with the detection of anti-CASPR2 antibodies in the blood and, with greater sensitivity, in the cerebrospinal fluid (CSF) being hallmark features [5].

Treatment strategies primarily focus on immunosuppressive therapies aimed at modulating the aberrant immune response. Corticosteroids, intravenous immunoglobulins, and plasma exchange are commonly employed modalities to alleviate symptoms and prevent disease progression. The response to initial immunotherapy is often favorable, but some patients remain severely disabled, requiring long-term immunotherapy [6].

## Case presentation

We report the case of a 70-year-old man with a previous history of type 2 diabetes mellitus, hypertension, hearing loss, azoospermia, and a history of smoking (60 pack-years). He was admitted to the emergency department with complaints of slurred speech and imbalance over the last five hours. He also reported a "pins and needles" feeling and occasional itchiness in his feet for the previous six months, along with a weight loss of 7 kilograms.

The neurological examination revealed slow and dysmetric saccades, severe dysarthria, exaggerated but symmetric deep tendon reflexes (except for C5-C6 and S1-S2) with bilateral Babinski sign, myokymias in the lower limb muscles and gastrocnemius myotonia, and a pancerebellar syndrome characterized by dysmetria in the finger-to-nose and heel-to-shin tests, and ataxic gait (widened base, impaired balance, and impaired tandem walking).

The stroke code was activated, and a brain computed tomography (CT) scan and computed tomography angiography (CTA), along with basic laboratory testing, were performed. No relevant findings were observed, and the patient spontaneously recovered after 14 hours. He was discharged the same day with the impression of transient ischemic attack under antiplatelet treatment with 100mg acetylsalicylic acid. Over the next four months, the patient experienced three similar episodes related to stressful events: witnessing his wife's car accident, preparing for a digestive endoscopy, and receiving the COVID-19 vaccine. During the last episode, he returned to the emergency department due to persistent symptoms (more than 24 hours) and was admitted for further evaluation.

The patient underwent comprehensive laboratory testing for treatable late-onset ataxias, with no relevant findings on the following tests: complete blood counts with differential, blood chemistries, renal and liver function tests, ammonia, thyroid function, vitamin levels (B12, B1, E), infectious serologies (HIV antibody, Lyme antibody, VDRL), serum protein electrophoresis, antinuclear antibodies (ANA), thyroperoxidase (TPO) antibodies, paraneoplastic antibodies, and antigliadin antibodies (IgA and IgG). Regarding diabetes control, the HbA1c value was 7.1% (NR: 3.4-5.8%).

**Figure 1:**
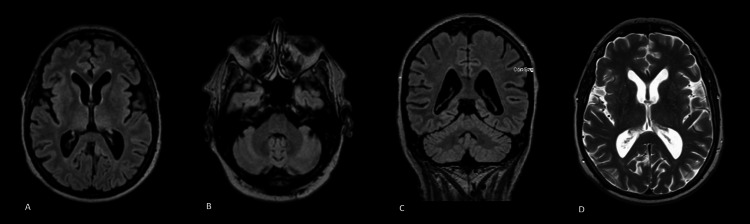
Brain MRI (Magnetic Resonance Imaging) study A- MRI axial FLAIR image: there are no focal areas of abnormal signal intensity or lesions identified. Both gray and white matter structures appear within normal limits, with no evidence of other pathological findings; B- MRI axial FLAIR image cerebellum: There is no evidence of cerebellar atrophy or other pathological findings;  C- MRI coronal FLAIR image: prominence of the cerebrospinal fluid (CSF) spaces and ventricular system, indicating increased CSF circulation pathways and a slight reduction in global brain volume, without clear lobar predominance; D- MRI axial T2-Weighted image: did not reveal any notable changes or abnormalities.

The cerebrospinal fluid (CSF) analysis revealed oligoclonal bands, with normal basic cytochemistry and a negative *Tropheryma whipplei *PCR. Anti-CASPR2 antibodies were positive in both serum and CSF. The remaining antineuronal antibodies were negative both in the blood and in the CSF. Electromyography (EMG) was normal, with no findings suggestive of peripheral hyperexcitability during the exam. Contrast-enhanced brain MRI revealed prominence of the cerebrospinal fluid (CSF) spaces and ventricular system, indicating increased CSF circulation pathways and a slight reduction in global brain volume (Figure 1).

**Figure 2:**
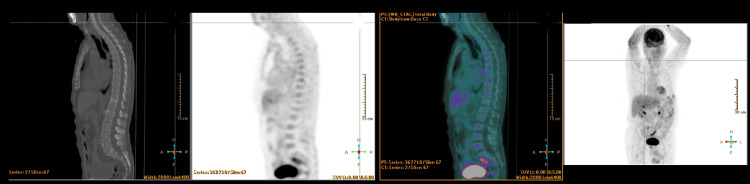
PET-18FDG (Positron Emission Tomography with 18F-Fluorodeoxyglucose) In the lung parenchyma, a small nodular formation in the upper left lobe, apparently calcified, likely related to a granuloma is noticeable. No clearly suspicious hypermetabolic lymph nodes are seen. Liver, spleen, adrenal glands, and pancreas show no suspicious hypermetabolic changes. Increased uptake of 18F-FDG is noted in the intestinal loops, likely of physiological nature. There is increased uptake of 18F-FDG in the lateral aspect of the 7th, 8th, and 9th left rib arcs, associated with traces of fracture. The remaining skeleton shows diffuse uptake of the radiopharmaceutical, with mild intensity and heterogeneous distribution. No other significant findings were noted. There were no functional changes suggestive of high-grade malignant neoplastic lesions with metabolic activity.

Paraneoplastic investigation with a thoraco-abdominal-pelvic CT scan and positron emission tomography (PET) with 18F-Fluorodeoxyglucose scan did not find any suspicious lesions of malignancy (Figure 2). The patient also underwent endoscopy, colonoscopy, and duodenal biopsies to investigate *Tropheryma whipplei*, thyroid ultrasound, and testicular ultrasound, all without pathological findings. Next-generation sequencing panel for episodic ataxias revealed no mutations.

The patient underwent treatment with a three-day course of intravenous methylprednisolone (1000mg/day), followed by plasmapheresis (seven sessions every other day), with a good response but without complete resolution of the symptoms. Intravenous immunoglobulins were introduced one week later due to persistent imbalance and neuropathic pain in the lower limbs. He remained hospitalized for one month without further episodes and showed continuous improvement of residual symptoms. At the time of discharge, he still had mild dysarthria and intention tremor.

Over the two-year follow-up, the patient continued to receive monthly intravenous immunoglobulin treatments in three-day cycles. He ultimately started treatment with rituximab every six months due to residual neuropathic pain and episodes of ataxia (which usually lasted for 2-3 hours) related to stressful events. In the following months, the patient was also diagnosed with mild cognitive impairment and depression, likely as part of the immune-mediated syndrome.

## Discussion

Our patient presented with recurrent, self-limited episodes of sudden cerebellar ataxia. After extensive investigation, he was diagnosed with a syndrome associated with anti-CASPR2 antibodies. There was a clear association between stress factors and the onset of the ataxic episodes. To date, there is no evidence of concomitant neoplasm, even after a second paraneoplastic investigation conducted one year after the diagnosis.

Unlike other cases reported in the literature, there were no clear cerebral signs at presentation, and both the brain MRI and EMG were unremarkable. The presence of slurred speech, gait imbalance, and limb dysmetria during these episodes strongly indicates cerebellar involvement.

While permanent ataxia is a recognized feature of anti-CASPR2 antibody-associated encephalitis, only a few case reports describe episodic ataxia in patients with autoimmune encephalitis. The similarity between the paroxysmal ataxia experienced by patients and the symptoms seen in inherited channelopathies like episodic ataxia (EA) is remarkable. Notably, neuromyotonia can occur in individuals with EA type 1 and anti-CASPR2 antibodies, indicating comparable ion channel abnormalities in both conditions [7,8]. Some authors suggest that transient cerebellar ataxia should be considered as part of the array of symptoms associated with anti-CASPR2 antibody-related conditions. The resemblance of the paroxysmal symptoms to those seen in hereditary channelopathies suggests a potential involvement of ion channel dysfunction in the pathogenesis of syndromes linked to anti-CASPR2 antibodies [9,10].

The patient responded partially to plasma exchange treatment, and the most disabling symptoms (gait ataxia and slurred speech) were successfully controlled with monthly intravenous immunoglobulin. The residual neuropathic pain and short-duration episodes of ataxia were ultimately treated with rituximab every six months, with a good response, as supported by other studies [11].

## Conclusions

The neurological manifestations that led to the diagnosis of anti-CASPR2 antibody-associated syndrome were intermittent, self-limiting episodes of ataxia, often triggered by concurrent stress-inducing factors such as emotional stress, prolonged fasting, and vaccination. This case supports suggests that paroxysmal cerebellar ataxia must be included in the spectrum of anti-CASPR2 antibody syndrome. It also highlights the need to consider immune-mediated causes, particularly anti-CASPR2, in patients with episodic ataxia, even if they do not present with classical signs like cerebral dysfunction or peripheral hyperexcitability.
